# Adapting Metacognitive Therapy to Children with Generalised Anxiety Disorder: Suggestions for a Manual

**DOI:** 10.1007/s10879-015-9294-3

**Published:** 2015-01-23

**Authors:** Barbara Hoff Esbjørn, Nicoline Normann, Marie Louise Reinholdt-Dunne

**Affiliations:** Department of Psychology, University of Copenhagen, Øster Farimagsgade 2A, 1353 Copenhagen K, Copenhagen, Denmark

**Keywords:** Metacognitive therapy, Children, Generalised anxiety disorder, Disorder-specific treatment

## Abstract

The metacognitive model and therapy has proven to be a promising theory and intervention for emotional disorders in adults. The model has also received empirical support in normal and clinical child samples. The purpose of the present study was to adapt metacognitive therapy to children (MCT-c) with generalised anxiety disorder (GAD) and create suggestions for an adapted manual. The adaptation was based on the structure and techniques used in MCT for adults with GAD. However, the developmental limitations of children were taken into account. For instance, therapy was aided with worksheets, practical exercises and delivered in a group format. Overall, the intervention relied heavily on practising MCT techniques in vivo with therapist assistance. A detailed description of how the manual was adapted for this age group is given, and examples from a group of four children are presented in a case series. Findings indicate that the adapted version of the metacognitive techniques and manual for children is feasible.

## Introduction

As researchers in the field of childhood anxiety, we attempt to improve our theoretical understanding and treatment efficacy for the benefit of youth suffering from anxiety disorders. Most treatment manuals are currently based on cognitive behaviour therapy (CBT), which is a well-established and effective treatment. The percentage of children who become free of all anxiety disorders after CBT is estimated to be 59 % (James et al. 2013). Within-group effect sizes range from *d* = 0.74 for child self-report to *d* = 1.06 for parent-reported anxiety decreases (Ishikawa et al. 2007). Although results are encouraging, we must acknowledge that approximately 40 % of children receiving CBT do not respond sufficiently. This highlights the need for improvement in our treatment approach. Within the childhood literature, several attempts have been made to improve CBT programs. For example, some have added a family component, investigating if family CBT would be superior to individual CBT. However, findings are equivocal, and a firm positive effect of including parents has yet to be established (Breinholst et al. 2012).

### Improving Treatment Outcomes

A different approach to improving outcomes is to examine if the theoretical models and corresponding treatments in the adult literature may be applicable to childhood samples. With respect to generalised anxiety disorder (GAD), the metacognitive model (MCM) and therapy has received substantial empirical support and promising treatment results for adults (e.g. Van der Heiden et al. 2012; Wells 2013; Wells et al. 2010). The MCM and treatment of GAD was developed by Wells (1995). The approach is based on transdiagnostic principles that emotional disorders arise as a result of a Cognitive Attentional Syndrome (CAS; Wells and Matthews 1996). The CAS is defined as an inward direction of attention, repetitive negative thinking (i.e., worry/rumination) and coping strategies that maintain anxiety (e.g., threat monitoring and thought suppression). In persons suffering from GAD, CAS is related to the presence of positive and negative metacognitive beliefs about worry (Wells 1995, 2009). The MCM of GAD states that it is metacognitive beliefs about the uncontrollability and dangerousness of worry that cause the disorder rather than the content of the worrisome thoughts (Wells 1995). This model has been supported by several studies (for a review, see Wells 2009).

### Metacognitive Therapy for Adults

Core components of metacognitive therapy (MCT) for GAD include detecting the positive and negative metacognitive beliefs about worry and challenging these both verbally and via behavioural experiments, for example by practicing the postponement of worries or trying to lose control of them. The patient is also helped to reduce the CAS in response to negative thoughts that normally trigger worry. Detached mindfulness (DM) is introduced as a new way of responding to such triggers. DM refers to the ability to acknowledge the existence of thoughts, and at the same time distance oneself from them without reacting or responding to them. MCT for GAD has been included in the NHS NICE guidelines for GAD (NICE 2012), and studies indicate that MCT may have a larger effect compared to CBT (Normann et al. 2014; van der Heiden et al. 2012). Thus, a metacognitive approach to childhood GAD may be a promising path to explore.

### The Metacognitive Model in Children

The first step in applying this model to children is to investigate if it is developmentally appropriate. Only if children have achieved the required cognitive level and skills may the model and corresponding treatment be applicable. Recently, such studies have been conducted in childhood and adolescent samples. A review of the literature concluded that the application of MCM to children with GAD is promising (Ellis and Hudson 2010). The developmental literature supports that children possess the cognitive skills involved in the MCM from early school age years. Some of these skills, e.g. knowing that attention is selective and limited, develops between the ages 5 and 8 years (Pillow 2008). Knowing when and how you came to know something develops at 6 years of age (Flavell 1999). Furthermore, children endorse negative and positive beliefs about worry. They hold beliefs that worry is difficult to control (Muris et al. 1998), that it may have a negative influence on wellbeing, but also that it may help them by thinking things through or keeping safe (Wilson and Hughes 2011).

Existing studies of the relation between anxiety, worry and metacognitive beliefs in clinical compared to normal samples have provided mixed results. Some studies found no significant difference in level of negative beliefs about worry held by clinical and non-clinical youth (Bacow et al. 2009), nor between youth with GAD compared to other anxiety disorders (Bacow et al. 2010). Other research groups reported that clinically anxious youth endorsed elevated levels of both positive and negative metacognitions compared to non-clinical controls (Ellis and Hudson 2011; Smith and Hudson 2013). Adolescents with GAD did however not differ from adolescents who had other types of anxiety (Ellis and Hudson 2011). Finally, a recent study that compared metacognitions in 7–12 years old clinically anxious children and non-clinical controls found strong support for the MCM. Children with GAD endorsed higher levels of unhelpful metacognitive beliefs than children with other types of anxiety and non-clinical controls (Esbjørn et al. 2014). Overall, the MCM has received sufficient support in clinically anxious children to warrant an examination of how MCT could be adapted for children with GAD. The purposes of the current study were to (1) adapt and refine a group-based metacognitive therapy for children (MCT-c) with GAD, and (2) to report initial feasibility from a pilot case series study of this adaptation. A key uncertainty was if MCT could be applied in a developmentally appropriate format.

## Methods

### Design

The case series provides information on the first four children who participated in a project on the development of a group based MCT-c. The children were assessed pre- and post-treatment and at 6 months follow-up. We provide results from child and parent reports of internalizing symptoms at the three assessment points. We present and discuss the adjustments made from adult MCT to obtain a manual of MCT-c in a group format which was used with the four children. We also present the final manual which was adjusted according to the experiences from the case series.

### Measures

The Revised Child Anxiety and Depression Scale (RCADS; Chorpita et al. 2000) consists of 47 items, assessing DSM-IV symptoms of social phobia, generalised and separation anxiety disorder, panic disorder, obsessive compulsive disorder and depression. It is scored on a 4-point scale (0 = *never*, 1 = *sometimes*, 2 = *often* and 3 = *always*). The psychometric properties of the Danish version are satisfactory with good internal consistency and adequate reliability and validity Esbjørn et al. 2012). Both parent and child reports were used. The internal consistencies as measured by Cronbach’s α were 0.93, 0.86 and 0.72 for child, father and mother, respectively. A composite score was created for the parents’ report of their child’s internalizing symptoms. In one case, only one parent filled out RCADS at follow-up, and hence this data was used.

The Anxiety Disorders Interview Schedule for DSM-IV, Child and Parent Versions (ADIS-IV-C/P; Silverman and Albano 1996) consists of two independent parent and child interviews regarding DSM-IV symptoms of anxiety disorders and possible comorbidity. A clinical severity rating (CSR) ranging from 0 to 8 is given to determine the severity of the disorder. A score of ≥4 indicates clinical levels of difficulties.

### Participants

Children were referred by their parents to our university clinic for treatment. Inclusion criteria were: (1) a primary disorder of GAD according to both child and parent on the ADIS-IV-C/P; (2) age 7–13 years; (3) IQ screening ≥70 on picture completion, block design, vocabulary and information from WISC-III (Wechsler et al. 1991); and (4) one parent was native Danish.

#### Participant 1

Participant 1 was an 11-year-old Caucasian girl, with a full scale IQ of 86. She had previously received psychotherapy for her anxiety. Her parents were not cohabiting, and she lived primarily with her mother. The family was lower middle class. Her mother had received treatment for an eating disorder in her youth, and her father for substance abuse. The CSR of participant 1’s GAD was 8. Her worries were specifically related to school, achievement, perfectionism, health and disasters. She was comorbid with separation anxiety disorder, social phobia and specific phobia for illness. Although she did not meet diagnostic criteria, she also endorsed problems with her conduct.

#### Participant 2

Participant 2 was a 12-year-old Caucasian boy, with a full scale IQ of 84. He and the family had previously received psychological counselling. His parents were not cohabiting, and he moved between both parents. The family was higher middle class. The mother had received psychotherapy for low self-esteem. The CSR of participant 2’s GAD was 5. His worries were specifically related to school, achievement, economy, and social issues. He did not have any comorbid disorders. Although he did not fulfil diagnostic criteria, he endorsed problems with attention, activity levels and impulsivity.

#### Participant 3

Participant 3 was an 11-year-old Caucasian girl, with a full scale IQ of 127. She lived with both biological parents. The family was lower middle class. Her mother had received psychotherapy for anxiety for a minimum of 1 year. The CSR of participant 3’s GAD was 6, and her worries were specifically related to achievement, perfectionism, and health of others. She was comorbid with a specific phobia for vomit.

#### Participant 4

Participant 4 was an 11-year-old Caucasian boy, with a full scale IQ of 99. He lived with both his biological parents who had no known psychopathology. The family was higher middle class. The CSR of participant 4’s GAD was 8. His worries were specifically related to getting a normal life, school, health of others, achievement and perfectionism. He was comorbid with specific phobia for loud noises, social phobia and separation anxiety disorder. Prior to participation in our project he had been day admitted to a child psychiatric hospital for 8 weeks due to depression and anxiety. Before discharge, he had been put on a low dosage medication (Fontex) with a plan to increase to effective levels. This increase was put on standby during participation in our project, thus the dosage remained stable across therapy. Medical treatment was terminated post treatment. He had not attended regular school prior to day admission.

### Procedure

Parents gave written informed consent for their own and their child’s participation in the study, and the children gave assent to participation. The study and data collection was approved by the Institutional Ethical Review Board of Department of Psychology, University of Copenhagen. Families were assessed prior to, after treatment and at 6 months follow-up. The posttreatment assessment took place after the booster session. Families filled out the questionnaires at home, and the ADIS-IV-C/P was administered at the clinic by trained psychology students or clinical staff who were blinded to the intake diagnoses of the child. Throughout the project period supervision was provided to testers to ensure reliability of the diagnoses. A specialist in clinical child psychology examined the videos, and consensus agreement was obtained on cases where comorbidity made judgements of diagnoses difficult. Three female therapists provided the group therapy. Two were authorized clinical psychologists; one of these being a specialist in psychotherapy, and one was a master-level student. The therapists received supervision from the originator of the therapy to ensure that the principles of MCT were applied correctly.

### Treatment

#### General Considerations in Adapting MCT to Children

The development of MCT-c was based on the structure and general outline of the treatment plan for adults with GAD (Wells 2009). However, downward extensions of adult treatment programs must be adjusted to the specific needs of children (Spence et al. 2008), as they lack the social, linguistic and cognitive sophistication that unmodified treatment techniques require (Reinecke et al. 2002). Many children find it difficult to generalise knowledge gained during verbal therapeutic conversations to real-life situations with elevated anxiety levels (Stallard 2009). Socratic dialogue may assist the child in the process; however, the child will often respond with “I don’t know”. Prompting assisted with visual cues, work sheets and practical exercises, as well as group treatment, where the child can observe other children with different experiences may increase the child’s awareness of their metacognitions, thoughts and emotions. This may increase the child’s motivation for the therapeutic task. In the present case series, these factors were taken into consideration. The child sessions consisted of a mixture of psycho-education, group discussions attempting to engage the children in Socratic dialogue, use of work-sheets, pictures, metaphors (unhelpful metacognitions being like old computer software, we need to upgrade the system) and experiments. Metacognitions were identified and challenged, and techniques were practiced in vivo. Socialisation to the CAS was made developmentally appropriate by an illustration of a child with a glass bubble closing in around him, being overly attentive to thoughts and bodily feelings, being so occupied that he does not register all the other aspects of the environment outside the bubble. In contrast to MCT for adults, our adaptation for children relied heavily on practising MCT components in vivo with therapist guidance. The treatment applied in the case series study consisted of two individual family sessions, two parent group workshops, ten child group sessions and one booster session.

#### Adapting Specific MCT Components to Children

*Attention Training* Although typically not part of MCT for GAD, attention training was included in MCT-c for two reasons. First, it increases awareness that thoughts are like noise. The child can choose not to react to them. Second, it teaches the child that he may take voluntary control over his attention. The children practiced acknowledging that attention sometimes slips, but can voluntarily be redirected to the selected stimuli. A parallel was drawn between attention slipping to an irrelevant stimulus and responding to intrusive thoughts, suggesting that this is a habit, rather than an uncontrollable process. As with adults, the training included selective attention, rapid attention shifting and divided attention. An audio file was created for the children to practice at home. Attention training was conceptualised as a mental workout for the brain that will help interrupt the self-focused attention in CAS. Attending voluntarily to selected stimuli in the environment, while leaving worries alone (situational attentional refocusing), was applied as the first-choice coping mechanism by some of the children in anxiety and worry provoking situations.

*Detached Mindfulness and Challenging Negative Metacognitive Beliefs* DM involves to notice thoughts that trigger worry, but to leave them alone without responding to them. It may be applied to challenge the belief that worries are uncontrollable. DM was explained as a new way of responding to thoughts, and several metaphors were used in order to illustrate the rationale behind the technique. One metaphor was the train metaphor. It illustrates that it is your own choice whether you want to engage in a trigger thought or leave it alone. When you see a train entering the station, you can get on it, but you can also choose not to. If you wait and do nothing, it will move along, which is analogous to a triggering thought. A field trip to the local subway station was conducted for the children to see how trains moved along similar to thoughts. The outing provoked fears and worry, and this provided opportunities to practise DM in vivo, and gave therapists the opportunity to ensure that techniques were applied correctly. In line with the train metaphor, we explained that you cannot force the train to move, you have to wait until it drives off in its own tempo. Children received cue cards to remind them of the DM metaphors.

To increase the use of DM, homework included applying DM to triggers and postponing worry until a certain time of the day. The postponement of worry was used to further challenge beliefs regarding lack of control; i.e. “if you have no control, how were you able to postpone the worry then?”. Finally, negative metacognitive beliefs that worry can make you ill were challenged. The beliefs were elicited with help from worksheets where children could cross off metacognitive beliefs that were true for them. Individual experiments were planned where the children would conduct anxiety-provoking tasks investigating if their worry would make them ill or go crazy. These included trying to worry as much as possible and investigate what happened, and interviews of strangers to investigate if they held metacognitive beliefs that worry would make them ill.

*Challenging Positive Metacognitive Beliefs* Positive metacognitive beliefs include that worry is helpful and prepares you for future events. In MCT-c these were elicited by worksheets. Following psychoeducation on how positive beliefs contribute to maintain worry, these were challenged using the worry-mismatch strategy (Wells 2009). Children wrote down individual worry and reality scripts to investigate if worry is useful. Both retrospective scripts, based on a recent episode of worry, and prospective scripts about near future events were made. Experiments were carried out in session and the homework was to test whether worrying was helpful in predicting what actually happened.

#### Involving parents in MCT for children

One must consider if and how to involve parents in their child’s treatment. Studies on parent–child relations suggest that parental over-involvement and intrusiveness are related to anxiety as it reduces the child’s experience of control (McLeod et al. 2011). From an MCT perspective, perceived lack of control over internal events such as worry, may maintain the belief that worry is uncontrollable and can only be stopped by seeking reassurance from parents. We therefore addressed such processes in the parent–child relation.

Parent workshops were conducted prior to and halfway through child treatment. Individual family sessions were conducted halfway through therapy and after the tenth child session. The first workshop included psychoeducation on GAD and socialisation to the MCM. We discussed how CAS behaviours were likely to maintain worry. Therapists moderated the discussions and identified behaviours that may be helpful in the short term (e.g., avoidance or reassurance seeking), but be negative in the long term. For example, most parents are very engaged in their child’s worries and often reassure their child that they would not happen in reality. We explained how analysing the probability of worries and giving worries attention becomes a maintaining factor of CAS, and discussed alternative ways of supporting their child, e.g. by telling the child that they were to try not to give their trigger attention. As children started to experience more control over their worries, part of their homework became to let go of maladaptive coping behaviours, such as calling parents to check if they are alright, seeking reassurance or avoiding situations that could trigger worry. The second workshop and the individual family sessions consisted of discussions about the model, techniques and progress or lack of progress in their child’s therapy.

## Results

All four children and their parents completed the course of therapy, suggesting that the intervention was acceptable. At posttreatment, participants 2, 3 and 4 were free of all anxiety disorders. Participant 1 continued to fulfil criteria for GAD, specific phobia and social phobia; however, the CSR of GAD had dropped from 8 to 5. At follow-up, participant 1 continued to meet criteria for specific phobia. Participants 2 and 3 were free of all disorders. Follow-up data for participant 4 is missing. Three months following posttreatment, participant 4 had changed school and experienced difficulties with peer relations. His mother was very anxious that her behaviour would cause him to experience a severe relapse. Therefore he and his mother received individual treatment outside the current project; participant 4 received social skills training. This treatment took place during the follow-up period, resulting in lack of follow-up data for participant 4.

**Fig. 1:**
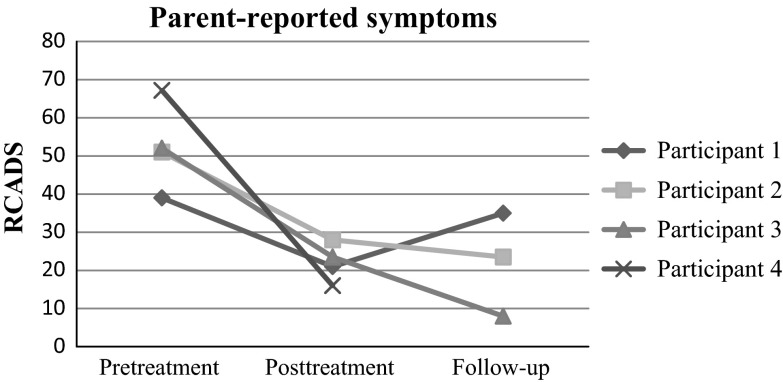
Parent-reported symptoms

**Fig. 2:**
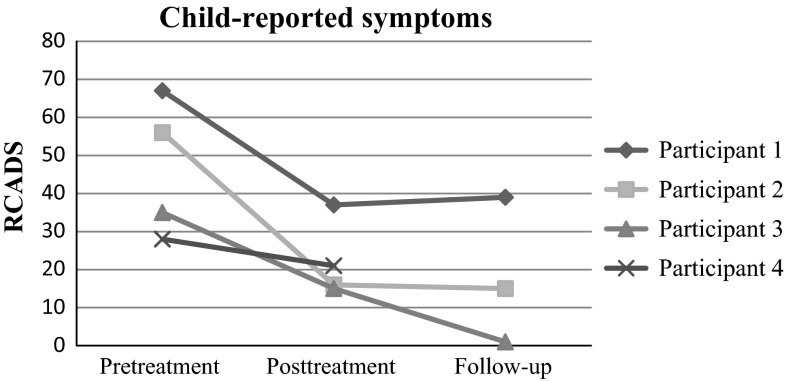
Child-reported symptoms

Figures 1 and 2 display parent- and child-reported internalizing symptoms as measured by the RCADS across time. Despite some differences in parent- and child report, a similar tendency of a decline in symptoms from pre- to post-treatment is observed for all cases, although there was a marked discrepancy between parent- and child-report for Participant 4. We did not find any systematic change from posttreatment to follow-up.

### Clinically Significant Change

We calculated Reliable Change Index (RCI; Jacobson and Truax 1991) to examine clinically significant change. RCI was computed from RCADS-C based on a community sample of 658 Danish children (unpublished data). This revealed a RCI of 9.87. Participants 1, 2 and 3 had clinically significant improvements from pre- to post-treatment. Participant 1 reported a drop of 30 points on the RCADS-c, despite still fulfilling diagnostic criteria. Participant 4 did not report clinically significant change. From pretreatment to follow-up clinically significant change was also observed for participants 1, 2 and 3.

### Reaching a Final MCT-c Manual

Our experiences with the four children led to adjustments to the manual. First, we eliminated the individual family sessions. As parents engaged in the workshops and shared their understandings and concerns relatively freely, individual family sessions became a mere repetition. Furthermore, the amount of techniques provided became overwhelming and some were never applied by the children. We reduced the number of therapy sessions, and focussed on selected metaphors and components that appealed to the children. The most commonly applied techniques were DM metaphors (worry as trains or clouds, imagining yourself being in a helicopter looking down at yourself and your worries, writing your worry trigger on the window, looking at it or out on the world), situational attentional refocusing, practicing not to ask parents for reassurance, and seeking out situations triggering worry, while applying DM. The final manual consisted of two parent workshops, eight child group sessions and one voluntary booster session for both children and parents (3–5 weeks after termination of group sessions). All sessions lasted 2 h.

### Child Sessions

The aims, components and homework of each of the child sessions are described below. The *first* session aims to normalize symptoms and familiarize the child to the metacognitive approach. Children share their worries, are socialized to MCM and CAS and are introduced to the difference between triggers and worries. Homework consists of a worksheet on which the child registers triggers eliciting worry. The *second* session aims at challenging beliefs that attention is uncontrollable. We discuss triggers versus worries, introduce the rationale for, and practice attention training in session. Homework consists of listening to the attention training audio file and register self-attention before and after having done so. The *third* session aims at practicing attentional flexibility and challenging the belief that worry is uncontrollable. We identify negative beliefs about uncontrollability and introduce DM using free association and writing/drawing triggers on a window. Homework consists of applying DM and postponing worry to a specified time (maximum 15 min) during the day. The *fourth* session continues to challenge uncontrollability beliefs. We go on an outing to the local subway station and watch trains pass by in their own tempo. This provides an opportunity to practice (and problem shoot) DM and situational attentional refocussing while worrying. Homework is similar to that of the third session. The *fifth* session continues to challenge uncontrollability beliefs. This is done by playing a board game which we developed. This helps children practice to voluntarily stop responding to triggers. Homework is as in previous sessions, only if no worries are experienced in their daily lives, situations eliciting worries should be sought out and DM practiced. The *sixth* session aims at challenging danger beliefs and exploring maladaptive coping strategies. We identify danger beliefs and coping strategies as avoidance, reassurance seeking and thought suppression. These are challenged in session using experiments. Homework consists of applying DM in situations that would normally have been avoided. The *seventh* session aims at challenging positive beliefs about worry. We work with worry and reality scripts. During the session we test if worry is useful or not. The homework is to conduct a prospective worry versus reality script in order to test out whether worry is helpful. The *eighth* session aims at preventing relapse. Old plans/strategies versus new plans/strategies and prevailing triggers are identified. Also therapy completion is celebrated. Homework consists of implementing the new plan and continuing to seek out situations eliciting difficult triggers.

### Parent Workshops and Voluntary Booster Session

The parent workshops are conducted prior to the 1st and 5th child session. The aims of these are to familiarize parents to the metacognitive approach and increase awareness of how CAS is maintained. The components include socialization to the model and CAS and discussions about CAS-related maintaining factors. In the second workshop we further present CAS mechanisms and discuss how parents can apply effective coping strategies in managing their child’s worry. The voluntary booster session aims at reinforcing the relapse prevention plan. It consists of an update of old versus new plans that are discussed with the parent and child. Homework is to continue to apply the new plan.

## Discussion

A key uncertainty in our project was whether we would be able to adapt MCT for children. Although the literature provides growing evidence for the application of the MCM in youth (Ellis and Hudson 2011), it remained a challenge to identify (1) which elements needed adjustment and (2) how adjustments could be made without changing the fundamental emphasis and message of MCT. Generally, we experienced that children complied with the therapy by completing assignments in session and as homework, and practicing the techniques. This clinical impression is supported by lack of drop out of therapy and a decline in anxiety during treatment.

Our previous knowledge of clinical child psychology assisted us in selecting age-appropriate approaches to the MCT components. One such adaption is the addition of attention training and the corresponding in vivo training of flexible, voluntary attention shifting in real life situations. Although not part of the core manual for GAD (Wells 2009), practicing attention training was deemed a developmentally appropriate step towards achieving a metacognitive mode of experiencing. Children with GAD hold the metacognitive belief that worry is uncontrollable, and that this is also true for attention. As many of the children have comorbid disorders and typically do not differentiate between symptoms in their everyday lives, teaching different techniques also allowed the child to select the strategy that was most effective for their specific worries in any given situation.

### Issues in Developing the Final Manual

The manual applied in the present study was regarded as a pilot. Therapists met regularly in order to discuss if any changes were needed with regard to content and structure of the sessions. Our experiences with the group were discussed with a specialist in MCT, and several modifications were made. The adjustments were made to ensure high adherence to the MCT tradition and model as well as to the needs and abilities of children. An example hereof is that children are often less abstract in their thinking than adults. This was seen in one child who said that she used the cloud metaphor when worried. This metaphor is used to illustrate DM. It suggests to treat worry triggers like clouds passing along, to leave them alone and they will move on. Upon further questioning it turned out that the child, when worried, went outside and looked up into the sky and watched clouds until the worry had passed. This is a faultily application, where the metaphor becomes a means of distraction. The example illustrates two key issues: (1) Children tend to be concrete. Therefore child therapists must understand normal developmental variations to be aware of potential pitfalls in the children’s understanding and application of MCT techniques in order to catch and correct misunderstandings and maintaining behaviours. (2) The distinction between adaptive and maladaptive metacognitive coping strategies is a key issue for successful MCT. Clients tend to use CAS-based coping mechanisms including distraction and suppression of thoughts. Research indicates that these approaches are counterproductive in the long term as they enhance intrusive thoughts (see review by Wells 2009). Therapists must be particularly cautious in regard to socialisation and teaching of MCT techniques. Thus, the exploration of the child’s understanding following the application of these techniques is essential to ensure that conceptual fidelity is maintained.

## Limitations

The main limitation of the study was the small sample, but it was not intended to provide statistics that could be used for subsequent sample size estimates. Rather it was exploratory in nature and intended to provide preliminary information on the adaptation process of MCT techniques and examine the feasibility of applying MCT-c to children with GAD. Our study thus does not provide information on the efficacy of MCT-c. A second limitation was that none of the therapists were formally trained in MCT. To increase the likelihood of adherence to MCT principals, we therefore discussed the manual and our therapy with the originator of the therapy. Third, we did not examine how IQ impacted therapy, and it remains unknown whether IQ is influential on treatment outcome in MCT-c. Finally, our manual was disorder specific for children with GAD, although high levels of comorbidity are present in anxious children (Kendall et al. 2001). This is a potential limitation. As we have already included techniques developed for comorbid disorders, future research should investigate if MCT-c is applicable transdiagnostically. Research within the field of MCT for childhood populations is still in its infancy. Currently, manuals only exist for obsessive–compulsive disorder (Simons 2012) and GAD. It is therefore premature to suggest implications of MCT-c for clinical practice. However, it seems that MCT techniques can be applied in treating GAD in children. We suggest that the next step is to run a pilot evaluation of the efficacy of the final MCT-c manual.
